# Factors Contributing to *De Qi* in Acupuncture Randomized Clinical Trials

**DOI:** 10.1155/2013/329392

**Published:** 2013-05-30

**Authors:** Yi Yang, Lin-Peng Wang, Lei Zhang, Li-Chen Wang, Jia Wei, Jia-Jian Li, Yi-Le Sun

**Affiliations:** Acupuncture and Moxibustion Department, Beijing Hospital of Traditional Chinese Medicine, Capital Medical University, 23 Meishuguanhou Street, Dongcheng District, Beijing 100010, China

## Abstract

*De qi* is a core concept of acupuncture and is necessary to produce therapeutic effect. In 2010, *de qi* has been received as a term in the official extension of the CONSORT Statement. However, there are few articles that discuss which factors have influences on obtaining *de qi* in clinical trials. This paper aims to explore these factors and give advice on trial design in order to optimize *de qi* in acupuncture RCTs.

## 1. Background

One of the fundamental characteristics of traditional Chinese acupuncture is the “obtaining of *qi*,” more commonly known by the pinyin *de qi*. The *de* component means “obtain,” and the *qi* component is often translated as “vital energy” [1]. Commonly, the term *qi zhi* has been used for the same phenomenon, which indicates “arrival of *qi*.” Both concepts originated from the classic book of traditional Chinese medicine (TCM) named *Huang Di Neijing* (also translated as* Yellow Emperor's Inner Classic of Internal Medicine*), which is the first comprehensive literature of TCM and treated as the authoritative guideline for Chinese medication and acupuncture until present day [2].

*De qi* is considered by many acupuncturists to be a fundamental component of acupuncture since the early classical texts. *Neijing* (*Lingshu *Chapter 1) states “For acupuncture to be successful, the *qi *must arrive (*qi zhi*). Acupuncture's effects come about like the clouds blown away by the wind” [2]. A more famous saying from the acupuncture poem *Biao You Fu *(Ode to Clear Obscurity) reads “If the *qi* comes quickly, the effect will be quick. If the *qi* comes slowly, the effect will be bad” [3]. Consistently with the classics, recent studies also provide evidence in support of this *de qi* phenomenon and indicate a statistically significant correlation between *de qi* and treatment efficacy [4, 5].

According to the theory of TCM and acupuncture experts, *de qi* is a composite of variable sensations experienced by both the patient and the administering acupuncturist [6, 7]. Typically, the acupuncturist would perceive *de qi* as heaviness or tension on the needle while stimulating it. In response to being punctured, the patient would perceive a sensation of soreness, numbness, heaviness, distension around the point, or even a sensation travelling to a specific place [3, 8].

The randomized controlled trial (RCT) is considered the gold standard to provide evidence for a treatment's efficacy [9, 10]. To improve and standardize reporting quality of RCTs, the CONSORT Statement was published in 1996 and received extensive applications [11]. Subsequently, extensions to CONSORT have been developed to cover the reporting of nonpharmacological treatments and pragmatic trials [12–14]. Since there are acupuncture-specific aspects to reporting not covered by these extensions, the Standards for Reporting Interventions in Clinical Trials of Acupuncture (STRICTA) was published in 2001 and its revision was added as an official extension of CONSORT in 2010 [15]. In these guidelines, *de qi* was listed to be a necessary part in acupuncture interventions. However, on the STRICTA checklist, there is a lack of articles discussing which terms in this list have significant or potential influences on obtaining *de qi*, and thereby how to effectively utilize *de qi* to create valid acupuncture RCTs. This paper aims to explore these terms and give advice on their design methods in acupuncture RCTs.

Given that there is a broad diversity of acupuncture styles (e.g., traditional Chinese medicine, Japanese, Korean, Western, Five Element, ear acupuncture, etc.), this paper will focus only on traditional Chinese acupuncture due to the nature of the authors' clinical and research experiences.

### 1.1. Acupoints Selection

Acupoints vary in their ability and characteristic to produce *de qi*. Hui et al. found that among three commonly used acupoints LI4 (*Hegu*), ST36 (*Zusanli*), and LV3 (*Taichong*), LI4 showed the most prominent response in the frequency and intensity as well as the largest number of *de qi* sensations [16]. Cheng reported that acupoints on the head and extremities are more inclined to achieve *de qi* when compared with acupoints on the trunk [17]. Recent electrophysiologic studies explained these differences as a consequence of different nerve innervations and tissue structures around acupoints [17–19]. For example, they stated that stimulation to the trunk acupoints elicits somatic autonomic reflex at the spinal level whereas stimulation to the extremity acupoints elicits a somatic autonomic reflex at the brain level [19]. However, literature has pointed out that in comparison with daily practice, the needle sensation in experimental research is often required to be very strong in order to demonstrate a therapeutic effect [20]. Given the wide variety of acupoints' characteristics, acupuncture practitioners in future research should be flexible instead of seeking intense sensation of *de qi* blindly on every single acupoint. Excessive needle stimulation is prone to produce discomfort and noxious effects [21]. From another aspect, when selecting acupuncture points for noneffective control groups, those located in trunk or other thick body regions with fewer nerve fibers are considered to be the ideal choice. These acupoints are deemed to rarely elicit *de qi *and therefore produce only minor or no unexpected therapeutic effects.

In a study by Vincent et al., patients were asked to assess their needle sensations after acupuncture intervention when needles were applied to acupuncture points and nonacupuncture points (sham acupoints) [22]. The results showed no significant differences in needle sensations between acupuncture points and nonacupuncture points for any locations. Studies that shared similar results may be due to their inappropriate selection for sham acupoints. According to research about the nature of meridian and acupoint, we have recognized that acupoints are not as small as needle tips [23]. However, the upper range of them has not yet been established. Consequently, when selecting sham acupoints, the optimal distance away from real acupoints remains controversial. Sham acupoints which are too close to real ones may be included in the effective range of real acupoints and erroneously induce *de qi* in the control group. In order to provide more evidence for sham acupoint selection, further studies on the nature of meridian and acupoint range are required.

### 1.2. Depth of Insertion

The depth of insertion is another term that is directly related to the production of *de qi*. Park et al. applied acupuncture at four different depths including the epidermis, corium, fascia, and muscle [24]. Their results showed that only when punctured into the muscle can acupuncture produce statistically significant needle sensations (*de qi*). It has been demonstrated that many of the *de qi* sensations are conveyed by different nerve fiber systems. For example, sensations including aching, soreness, distension, heaviness, warmth, and dull pain are conveyed by the slower conducting *A*δ** and *C* fibers, whereas numbness is conveyed by the faster conducting *Aβ* fibers [25, 26]. Since these fibers are more densely distributed in the tendinomuscular layers [27], only acupuncture manipulation inserted into this depth could elicit valid *de qi* sensations. Based on these findings, superficial acupuncture without penetrating the skin is considered to be an ideal sham acupuncture intervention that effectively and easily blinds patients as well as avoids *de qi*.

### 1.3. Needle Stimulation

As major patterns of acupuncture manipulations, it has been confirmed that manual acupuncture and electroacupuncture (EA) produce different types and intensities of *de qi* sensations. For example, Leung et al. found that “aching” is the predominant *de qi* sensation derived from manual acupuncture whereas “tingling” is the predominant *de qi* sensation derived from EA [28]. One possible reason of this difference is that EA had different modulatory effects on the brain cortex from those by manual acupuncture [29]. It has been proven that different manual stimulation techniques (e.g., lifting-thrusting and twirling rotating) and variations in the direction, angle, and depth of needle insertion can affect the outcome of acupuncture treatment [30, 31]. However, there is no widespread agreement on how strong or which* de qi* sensations and needle stimulations should be used for a therapeutic effect.

Laser acupuncture usually serves as a contrast of therapeutic acupuncture, in the control group. Compared with a metal needle, laser acupuncture does not pierce skin and has always been considered to have no therapeutic effect as well as no evocation of *de qi.* However, in some study reports published in 2012, researchers have demonstrated that subjects with laser acupuncture also experienced *de qi* [32–34]. On the basis that *de qi* is the sign of acupuncture efficacy these studies signify that using laser acupuncture as an intervention in the control group may cause underestimation of acupuncture effects.

### 1.4. Practitioner Background

*De qi *is not only a fundamental sign of effective treatment, but also influences the speed of and potential for recovery [2, 5, 8]. Different technical processes of acupuncture insertion and stimulation lead to different characteristics of *de qi* and therefore different treatment results. As the person that conducts all of these manipulations, the acupuncturist is the vital determinant about the evocation and intensity of *de qi*. Hence it is essential to report pertinent information about the acupuncturist providing treatment including qualifications or affiliations and years in acupuncture practice, as well as any other experiences that may be relevant to the trial. Relevant differences (if any) in the qualification, training, and experience of the participating acupuncturists should be highlighted. Since the level of reporting has historically been poor, recent reviews of acupuncture trials have stressed the need to extensively document these characteristics [35, 36]. The eligibility criteria for acupuncturists should also be explained, as these will influence the homogeny of the trial results. Training for the acupuncturists before the trial is necessary and important for achieving uniform manipulations of needle insertion and stimulation to be conducted during the trial. Where there are known to be potential variations between practitioners, selecting a random sample of practitioners will reduce expertise bias and help improve the applicability of the results [37].

## 2. Discussion

The STRICTA checklist clearly states that, in acupuncture RCTs, eliciting of *de qi *as well as the difference between the responses required in the protocol and those actually obtained should be reported in the Results section [15]. But there is no mention in the statement of how to assess whether *de qi* was attained. The majority of acupuncture clinical studies only mention *de qi* at intervention design without any assessments or reports to describe whether the subjects and acupuncturists actually obtained *de qi *[36]. Since different experiences of needle sensations may be associated with different outcomes, it is absolutely essential that* de qi* is carefully considered. This is especially so given that these RCTs are evaluating the therapeutic effects of acupuncture with *de qi* being an important variable. The complexity of ensuring that similar levels of *de qi* were experienced is a major challenge in our field. For this reason, attempts have been made to apply a scale to quantify the needle sensation.

A number of researchers have sought to establish a credible rating scale for* de qi*, such as the McGill Pain Questionnaire [22], Subjective Acupuncture Sensation Scale (SASS) [4], the Massachusetts General Hospital Acupuncture Sensation Scale [38], the Southampton Needle Sensation Questionnaire [39], and the *de qi* composite [16]. It should be noted that the existing scales and questionnaires do have limitations. Some experienced acupuncturists have stated that the scale descriptors do not tally with those given by their patients who often described more subtle sensation, such as a cool flow sensation, or sensations going from one point to another [40]. Furthermore, the terms in some scales are not clear for patients to understand. For example, some patients could not easily identify the difference between the term is “spreading” and “radiating” [40]. However, given that *de qi* sensations have been recognized as an important variable for different individuals, conditions, and needle stimulations, the use of *de qi* scales will be a promising way to facilitate the *de qi* sensation being controlled for in clinical and experimental studies. In addition, quantitatively calculated *de qi* sensations may offer a meaningful method to further interpret the findings of RCTs and increase validity. It is also recommended that future studies should assess *de qi* at each acupuncture consultation to reduce recall bias. 

In TCM theory, the *de qi* felt by the acupuncture practitioner is as important as the *de qi* felt by the patient [6, 8, 41]. It is also reported that the practitioner's needle sensations are more objective than those of patients and are necessary for obtaining treatment results [42]. For some experts, *de qi* is an intuitive sensation that is affected by the condition of the patient or the anatomical location of the point and thereby hard to describe or assess in a quantitative or qualitative way. However, the practitioner questionnaire is considered to be a fairly reliable guide to the obtainment of *de qi *[43]. Even though there have been many attempts to assess the needle sensations experienced by the practitioner [44], a validated *de qi* questionnaire has not been developed. Further studies to develop tools assessing *de qi* based on the practitioner's feeling or senses should be conducted.

## 3. Conclusion

In recent years, although there has been increasing evidence from randomized trials and systematic reviews on the efficacy of acupuncture, the conclusions remain controversial. The lack of significant difference between real and sham acupuncture in RCTs may result from the omission of certain important components of acupuncture, especially the obtainment of *de qi*. According to the study reports of acupuncture, factors in the STRICTA statement including the point selection, needle stimulation, and depth of insertion will contribute to the achievement of *de qi*. Taking all these factors into consideration would have considerable implications for the design and interpretation of acupuncture clinical trials. Meanwhile, assessment of the *de qi* sensations for both patients and acupuncture practitioners may be conducive to ascertain and control the *de qi* sensations in future clinical and experimental studies.
